# Blood-Based Multi-Cancer Detection Using a Novel Variant Calling Assay (DEEPGEN^TM^): Early Clinical Results

**DOI:** 10.3390/cancers13164104

**Published:** 2021-08-15

**Authors:** Frederic Ris, Minia Hellan, Jonathan Douissard, Jorge J. Nieva, Frederic Triponez, Yanghee Woo, David Geller, Nicolas C. Buchs, Leo Buehler, Stefan Moenig, Christophe E. Iselin, Wolfram Karenovics, Patrick Petignat, Giang Thanh Lam, Manuela Undurraga Malinervo, Rebecca Tuttle, James Ouellette, Debashish Bose, Nael Ismail, Christian Toso

**Affiliations:** 1 Division of Visceral Surgery, Department of Surgery, University Hospital Geneva and Medical School, 1211 Geneva, Switzerland; j.douissard@gmail.com (J.D.); Nicolas.c.buchs@hcuge.ch (N.C.B.); stefan.moenig@hcuge.ch (S.M.); nael.physician.scientist@gmail.com (N.I.); christian.toso@hcuge.ch (C.T.); 2 Surgical Oncology, Wright State University, Dayton, OH 45435, USA; Minia.Hellan@ketteringhealth.org (M.H.); rebecca.tuttle@wright.edu (R.T.); jrouellett@premierhealth.com (J.O.); 3 Norris Cancer Center, Keck School of Medicine of USC, University of Southern California, Los Angeles, CA 90033, USA; jorge.nieva@med.usc.edu; 4 Division of Thoracic Surgery, Department of Surgery, University Hospital Geneva and Medical School, 1211 Geneva, Switzerland; Frederic.Triponez@hcuge.ch (F.T.); wolfram.karenovics@hcuge.ch (W.K.); 5 Division of Surgical Oncology, Department of Surgery and Cancer Immunotherapeutics Program, City of Hope, Duarte, CA 91010, USA; yhwoo@coh.org; 6 Department of Surgery, University of Pittsburgh, Pittsburg, PA 15260, USA; gellerda@upmc.edu; 7 Division of Visceral Surgery, Department of Surgery, University Hospital Fribourg, 1700 Fribourg, Switzerland; Leo.Buhler@hirslanden.ch; 8 Division of Urology, Department of Surgery, University Hospital Geneva and Medical School, 1211 Geneva, Switzerland; christophe.iselin@hcuge.ch; 9 Divison of Gynecology, University Hospital Geneva and Medical School, 1211 Geneva, Switzerland; Patrick.Petignat@hcuge.ch (P.P.); Giang.T.Lam@hcuge.ch (G.T.L.); Manuela.Undurraga@hcuge.ch (M.U.M.); 10 The Center for Hepatobiliary Disease, Mercy, Baltimore, MD 21202, USA; dbose@mdmercy.com

**Keywords:** liquid biopsy, DEEPGEN^TM^, cancer detection, early detection, cancer screening, cell-free DNA, machine-learning

## Abstract

**Simple Summary:**

Cancer remains a worldwide concern with significant burdens on the population and healthcare systems. Studies have shown that early detection is paramount in positive patient outcomes, although the standard of care screening is limited to a few cancers. When a tumor cell dies, it releases DNA into the bloodstream. This cell-free DNA can be extracted, and specific mutations identified. Technologies based on this principle are gaining popularity amongst physicians for cancer patients to inform personalized treatment. Additionally, if platforms are sensitive enough, blood-based multi-cancer screening can be performed. DEEPGEN^TM^ is a next-generation sequencing platform that has been optimized for early cancer detection. This study is a preliminary analysis of cancer detection rates across seven cancers using the DEEPGEN^TM^ platform.

**Abstract:**

This is an early clinical analysis of the DEEPGENTM platform for cancer detection. Newly diagnosed cancer patients and individuals with no known malignancy were included in a prospective open-label case-controlled study (NCT03517332). Plasma cfDNA that was extracted from peripheral blood was sequenced and data were processed using machine-learning algorithms to derive cancer prediction scores. A total of 260 cancer patients and 415 controls were included in the study. Overall, sensitivity for all cancers was 57% (95% CI: 52, 64) at 95% specificity, and 43% (95% CI: 37, 49) at 99% specificity. With 51% sensitivity and 95% specificity for all stage 1 cancers, the stage-specific sensitivities trended to improve with higher stages. Early results from this preliminary clinical, prospective evaluation of the DEEPGENTM liquid biopsy platform suggests the platform offers a clinically relevant ability to differentiate individuals with and without known cancer, even at early stages of cancer.

## 1. Introduction

Cancer is a major cause of mortality worldwide and a significant burden on patients as well as the healthcare system [1]. Major challenges include the absence of early diagnosis at a large scale, especially for average risk individuals because the standard of care screening only exists for a limited number of cancers for this population [2]. Thus, the current diagnostic pipeline is mostly empirical and primarily driven by an examination of the patient’s symptoms [2,3]. Due to delays in detection, most of the patients diagnosed with cancer miss the therapeutic window of opportunity to reset the disease’s trajectory [4,5]. Therefore, early detection using rapid, cost-effective, high-precision, and minimally invasive tools holds the promise to materially decrease morbidity and mortality in cancer [6,7].

Advances in sequencing technologies and associated genomic research have proven crucial to better understand the mutational patterns that accompany cancer [8,9]. Extensive molecular profiling of tumors has successfully led to the identification of key somatic mutations that drive transformation, evolution, and the continually adaptive behavior of cancer. Considering that dying cancer cells shed cell-free DNA (cfDNA) into the bloodstream, the ability to accurately detect these mutations broadly in the blood, especially at ultra-low variant allele frequencies (“VAF”, i.e., far below <1%), may provide an attractive alternative to standard tissue biopsy [10,11,12,13]. Indeed, blood-based biopsies—if designed appropriately for high sensitivity—can detect low prevalence mutations with a reduced signal-to-noise ratio (<0.1%), making them a potential high impact diagnostic tool for improving the precision of accompanying cancer treatments [10,11,13].

Nevertheless, blood-based biopsy, as with the current gold standard tissue biopsy, faces several challenges that must be overcome. One of the major drawbacks of blood-based biopsy is the low allele frequency of clinically significant variants, which often reside in the range of technical background noise [13,14]. This is especially true in early-stage cancer patients where the concentration of tumor DNA is low [13,14,15,16]. Moreover, the narrow base pair range of the fragments (140–200 bp), short life of DNA in the blood (1.5–2 h), tumor type, proliferation rate, and therapy create challenges for the ultimate utility of liquid biopsies for cancer screening [17,18,19]. Hence, extensive optimization of pre-analytical (e.g., extraction and storage) and analytical (e.g., bioinformatics and AI) processes is required to optimize precision. Until now, most commercially available platforms and those under pre-clinical validation have sub-optimal clinical performance [11,20,21,22]. Thus, there is a need for novel platforms that considerably improve detection accuracy and variant calling precision at ultra-low VAF to facilitate the detection of cancer, particularly in asymptomatic individuals. 

DEEPGEN^TM^ (Quantgene Inc., Santa Monica, CA, USA) is a newly developed full stack sequencing technology awarded with a Clinical Laboratory Improvement Amendments (CLIA) certificate. The platform addresses the common challenges of previous generation liquid biopsy platforms by combining yield-optimized cfDNA storage and extraction protocols, a broad panel assay, advanced error reduction chemistry, and ultra-deep next-generation sequencing (NGS) with a customized bioinformatics pipeline, highly scalable cloud software and innovative AI techniques to identify low-frequency variants accurately and consistently in the blood. Initial validation studies demonstrated that DEEPGEN^TM^ has high detection capabilities, capturing over 3000 mutations at VAFs down to >0.09%, and thus out-performs other publicly available platforms on a technical level [23]. Additionally, the mutations undergo a proprietary machine learning protocol that considers mutations and their VAFs to derive a prediction if cancer might be the underlying cause of the observation.

The objective of this pilot study is to evaluate the early clinical capabilities of the DEEPGEN^TM^ assay to discriminate individuals with yet-untreated cancers from individuals without a known cancer diagnosis.

## 2. Materials and Methods

### 2.1. Study Design and Participants

This is a preliminary analysis of prospective multi-center case–control, open-label pilot study (NCT03517332). Adult patients over 18 years of age with newly diagnosed and to that point untreated cancer were included in the cancer group. Patients with no known or previous history of malignancy were included in the control group. Written consent was secured before study enrollment. A representative set of patients with cancers of breast, lung, pancreas, liver, colon–rectum, prostate, or bladder ranging from stage I to IV and a control cohort were retrospectively selected to undergo processing and analysis. 

### 2.2. Sample Collection

A measure of 10–20 mL of venous blood was taken from the study participants using DNA stabilizing blood tubes (Streck, La Vista, NE, USA). Blood tubes were stored at room temperature and shipped to the location of DNA extraction.

### 2.3. DNA Preparation

Cell-free (cf) DNA was extracted and purified using Qiasymphony (Qiagen, Hilden, Germany) using a customized protocol that was designed to maximize extraction yield. Early samples were extracted manually. Extractions were transitioned to an automated process for most of the samples. DNA concentration was then determined using quantitative RT-PCR. Samples were stored at −80 °C until assigned for sequencing.

### 2.4. Sequencing

NGS libraries were prepared from cfDNA according to the manufacturer’s instructions (protocol based on QIAseq Targeted DNA Panel Handbook, R2; May 2017, Qiagen, Hilden, Germany) except for the DNA fragmentation. End-repair and Poly(A) tailing were followed by Illumina NGS adapter (Illumina, San Diego, CA, USA) ligation to cfDNA containing a sample index and a unique molecular identifier sequence (UMI). After UMI assignment, target enrichment of ligated cfDNA was performed by PCR using target specific DEEPGEN^TM^ primers. Library concentrations were determined with KAPA Library Quantification Kits for Illumina platforms (Roche Holding AG, Basel, Switzerland). Libraries were prepared using NovaSeq Reagent Kits (Illumina, San Diego, CA, USA) and sequenced with a 300-cycle S4 kit on a NovaSeq 6000 (Illumina, San Diego, CA, USA) with a mean raw sequencing depth of ~200,000×. 3062 genomic variants were targeted. All steps were carried out according to the manufacturer’s instructions.

### 2.5. Data Processing

FASTQ files were processed with the DEEPGEN^TM^ bioinformatics pipeline. Sequencing data from both paired-end reads were deployed, whereas information from the 2nd read was used to complement the sequence of read one. Each read was screened for the specific primer sequence and consensus sequences of the fragments were identified by consolidating reads based on primer and UMI information. Each unique consensus sequence was aligned to its reference via an optimized Smith–Waterman algorithm. Based on a whitelist with defined targets, single nucleotide polymorphisms (SNPs), multi nucleotide polymorphisms (MNPs), and short insertions/deletions (INDELS) (<50 base pairs) were recorded. Identical genomic alterations were summarized and the count coverage and resulting frequency (count/coverage × 100) for each unique variant were logged into a mutation table alongside their location and mutation information.

### 2.6. Machine Learning

The input data for the machine learning models consisted of mutation frequencies for the genomic targets computed by Quantgene’s bioinformatics pipeline. Samples with a mean sequencing coverage <200 across select targets were removed from the machine learning analysis. Random forest classifiers were trained via leave-one-out cross-validation to predict whether a sample belonged to the control or cancer cohort. For every sample, a random forest output of a predictive score with a continuous value between 0 and 1 indicated the likelihood of belonging to the cancer group in ascending order. A classifier was trained on all cancer and control samples as a full model. Cancer-specific classifiers only utilizing samples from a single organ of origin and all controls were trained for lung, pancreas, liver, colorectal, and bladder. The classifiers for prostate and breast cancer were limited to only male and female samples, respectively, for both control and cancer groups. Samples with manual and automated DNA extraction were analyzed separately.

### 2.7. Classification of Cancer Versus Non-Cancer (Predictive Score)

The cut-off values between a positive (cancer) from a negative (non-cancer) predictive score were derived for both a 95% and 99% specificity. Sensitivities for both specificity values were calculated for the full cancer model and organ-specific models. Cancer stage-specific sensitivities were calculated at 95% specificity.

### 2.8. Data Analysis

Machine learning models were trained and evaluated using the scikit-learn package (v0.24.0) in Python (v3.8.7, Python Software Foundation, Troisdorf, Germany) [24,25]. Results of the different cancer models adjusted for a 95% specificity were subjected to Kruskal–Wallis tests to screen for significant differences in sensitivity between cancer types and cancer stages, respectively. In case of a significant *p*-value (*p* < 0.05), a Wilcoxon rank sum test was performed as a post hoc procedure. Test were performed with R, version 4.0.2 (https://www.r-project.org, accessed on 25 June 2021).

### 2.9. False Positive Controls

If available, medical records of false-positive controls were assessed for relevant diseases that were present at the time of the liquid biopsy as well as during the follow-up until the date of review.

## 3. Results

### 3.1. Demographics

A total of 719 samples were sequenced, and of these, 44 were excluded from machine learning analysis due to a mean sequencing coverage <200 across select targets. The remaining 675 samples consisted of 25 bladder, 29 prostate, 30 lung, 27 liver, 40 pancreatic, 66 colorectal, 43 breast cancers, and 415 controls without known cancer diagnosis (controls). A total of 70 individuals with stage I (27%), 55 with stage II (21%), 73 with stage III (29%) and 27 cases with stage IV (10%) were included. The cancer stage remained clinically undetermined for 35 cases (13%). The mean age was 65.4 ± 10.9 years in cancers and 54.9 ± 15.5 years in controls (*p* < 0.001). There were 136 men and 124 women in the cancer group, and 117 men and 298 women in the control group (*p* < 0.001). A family history of cancer was found in 38% of the cancer patients and 44% of the control patients. A complete description of patients’ demographics can be found in Table 1.

### 3.2. Cancer Identification

**Figure 1:**
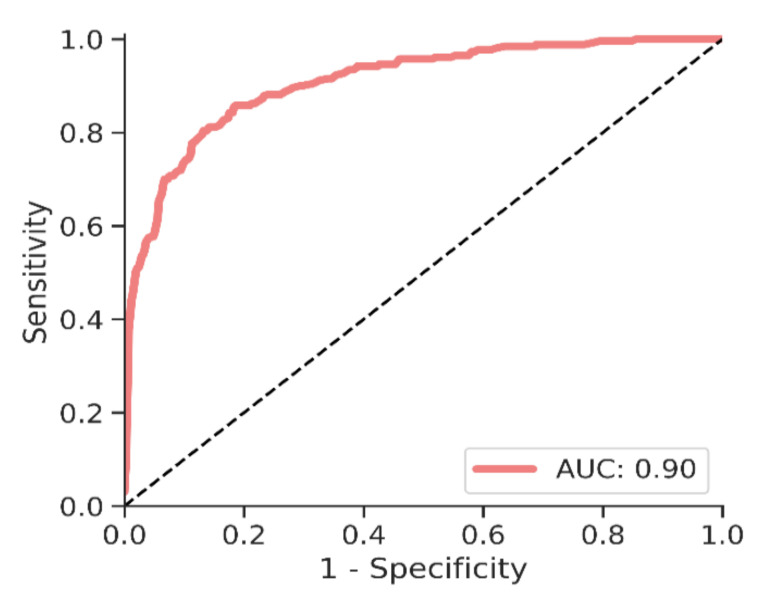
ROC curve for diagnosis of all cancers (full model).

The overall detection sensitivity in the machine learning model for all cancers was 57% (CI: 52, 64) at 95% specificity, and 43% (CI: 37, 49) at 99% specificity. The area under the curve (AUC) for this model was 0.90. A receiver operating characteristics (ROC) curve for the “all cancers” model is shown in Figure 1.

**Figure 2:**
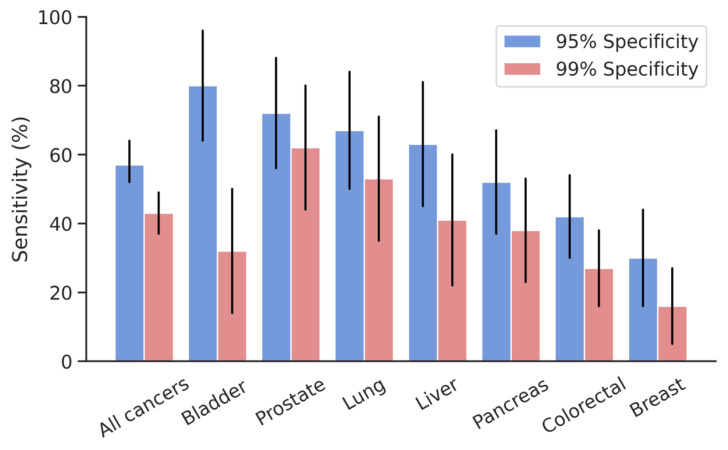
Sensitivity of DEEPGEN^TM^ platform by model at 99% and 95% specificity.

At 95% specificity, the individual organ models ranged in sensitivity from 30% for breast cancer to 80% for bladder cancer, with sensitivities higher than 50% for prostate (72% (CI: 56, 88) sensitivity), lung (67% (CI: 50, 84) sensitivity), liver (63% (CI: 45, 81) sensitivity), and pancreas (52% (CI: 37, 67) sensitivity). At 99% specificity, the individual organ models ranged in sensitivity from 16% for breast cancer to 62% for prostate cancer (*p* = 0.001). The bladder differed from the breast model (*p* = 0.0018) and the colorectal model (*p* = 0.0102), the breast model from the lung (*p* = 0.0120) and prostate (*p* = 0.0052) models, and the prostate model from the colorectal model (*p* = 0.0268). Detection performance for the “all cancer” and individual organ models can be found in Table 2 and Figure 2.

### 3.3. Impact of Cancer Stage

**Figure 3:**
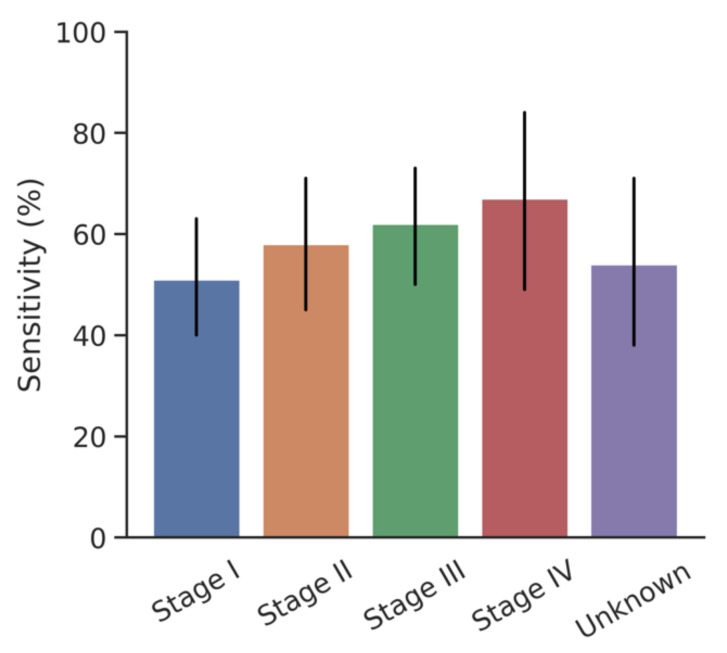
Sensitivity of DEEPGEN^TM^ platform by cancer stage at 95% specificity.

**Figure 4:**
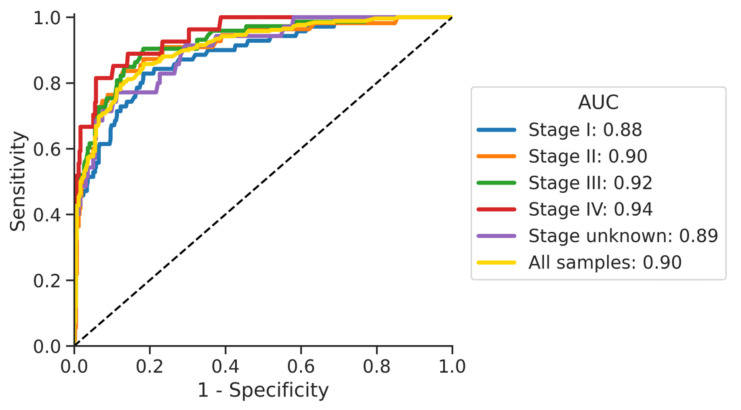
ROC curves for cancer stages.

At 95% specificity, the overall cancer sensitivity was 51% (CI: 40, 63) for stage I, 58% (CI: 45, 71) for stage II, 62% (CI: 50, 73) for stage III, and 67% (CI: 49, 84) for stage IV. Figure 3 shows the stage-specific sensitivities at 95% specificity (*p* = 0.5375) and Figure 4 shows the stage-specific areas under the curve.

The area under the curve (AUC) for the cancer stages ranged from 0.88 for stage I to 0.94 for stage 4.

### 3.4. False Positive Controls

The “all cancers” model resulted in 21 positives of the 415 controls at 95% specificity. Twenty-one medical records were available with a mean follow-up of 30.9 (±5.2) months. Three study controls were morbidly obese and scheduled for bariatric surgery. Four patients were hospitalized for an acute inflammation and two of those required emergency cholecystectomies for acute cholecystitis and acute cholangitis, respectively. Two individuals were diagnosed with a benign tumor at the time of the liquid biopsy and three were found with a benign lesion at 8-, 20-, 24- and 36-months and post-study inclusion (one patient developed two independent benign lesions).

## 4. Discussion

These early results of a comprehensive prospective evaluation of the DEEPGEN^TM^ platform suggest a clinically viable capability to differentiate individuals with and without cancer for seven different organs of origin. This finding was observed for a “full model”—including all assessed cancers as well as organ-specific models with varying ranges of performance for the different cancers. As such, it seems plausible to assume that the described liquid biopsy and data analytics innovations not only transpire to an excellent technical performance [23], but also into a relevant clinical capability. This finding is in line with similar projects in the field of liquid biopsy using different technological approaches [7,21,22,26]. Important differences in performance have occurred across cancers and have been described previously. A variety of reasons might cause this observation, including differences in tumor biology (less production of cfDNA of certain cancers), panel variants that do not equally cover all cancers, study logistics, including the time from blood draw to DNA extraction, and others. Further research is required to understand the details around this variability in outcomes per organ of origin.

While this is still a preliminary analysis of the first few samples of a larger specimen collection, the obvious question is how this promising technology can be utilized for an impactful clinical application beyond research. As outlined above, current population-based screening is insufficient and flawed, and leaves room to significantly improve cancer outcomes through earlier detection [2,6,27,28]. At the same time, any new screening tool must provide robust performance with a healthy balance of sensitivity and specificity to be applied for a population-wide clinical application. As such, and considering the above-described results, the DEEPGEN^TM^ platform surely holds promise for becoming an instrument for early cancer detection in a variety of applications. Of particular value is the demonstrated ability to adjust sensitivity and specificity for the full model and each organ-specific model. To focus on clinical usability and to reduce the number of false-positive results, the specificities have been set at 95% and 99%. In both settings, the sensitivity of DEEPGEN^TM^ is lower than for most standard of care screening methods and also considering the low sample size; this tool should not be used as a replacement for any kind of recommended population-based screening at this point.

However, there may be significant value in diagnostic tests that affect the Bayesian probability of cancer, regardless of underlying genetics. This may include the evaluation of unexplained weight loss in the elderly or other conditions in which the pre-test probability of malignancy is high such as low gradient ascites and exudative pleural effusions. Other high-risk populations may also benefit from these types of screening technologies including patients undergoing evaluation for solid organ transplants and those people who are exposed to high-risk carcinogens or excessive radiation. There may also be value in complementing current screening regimes that can potentially be filled by this technology, such as patients with LungRads-3 screening CT scans for lung cancer, BIRADS-3 mammograms, and following colonoscopy for the removal of polyps with dysplasia. As such, a low sensitivity and high specificity setting is most suitable for individuals at clinical inflection points where there are indeterminate findings, but where additional radiographic or pathologic explorations confer morbidity. The DEEPGEN^TM^ approach could reduce exposure to potential morbidity—physically and/or psychologically—caused by further diagnostics and anxiety. On the other hand, a high sensitivity and low specificity setting is most appropriate for any individual subject to standard of care screening. In that instance, the higher rate of false-positive results turns into a benefit in which a wider spread of currently recommended pathways is fostered. This spread of currently recommended pathways will include all their proven medical and economic benefits since these diagnostics would be the next logical step in confirming cancer suspicion [29,30]. As such, even at this early stage, liquid biopsy platforms such as DEEPGEN^TM^ can help to identify cancers that would otherwise not be detected, and at the same time, can improve cancer diagnostics by enforcing existing screening programs.

Besides this potential concrete application for personalized cancer detection, this technology should also be viewed as a more global diagnostic platform. As a matter of fact, just in the space of conventional oncology, the ability to find signs of cancer at the earliest possible moment opens several opportunities to improve patient care. Beyond aiding in diagnosing cancer early, mutations and mutational profiles found in the patients’ blood have demonstrated value in determining treatment success by assessing the presence of minimal residual disease (MRD), recurrence detection, and tumor typing to support treatment decisions for advanced disease [31,32]. Additionally, research might demonstrate the role of cfDNA for a range of diseases beyond cancer, including neurodegenerative and cardiovascular diseases; as such, it is plausible to assume that such platforms might transform into global tools of the modern physician’s armory.

All of these technologies, including DEEPGEN^TM^, are still at the beginning of their development and some important limitations exist to date. Besides the current overall performance, particularly the overlap of mutations across cancers, there is the problem of tissue-of-origin determination [33,34,35], and a liquid biopsy result that cannot firmly indicate the origin of cancer. As such, intelligent diagnostic algorithms must be developed and clinically studied before a widespread application in the screening sector can be imagined. In consideration of these potential challenges, the overall value of this technology needs to be studied in long-term clinical trials that analyze critical parameters, which include survival, quality of life, and costs. However, considering the promise of such technologies, significant investments should be made globally in the area of research and development. In the short- and mid-term, we foresee tremendous progress in all areas of this technology stack, ranging from DNA yielding, wet-lab technologies, and data analytics. In particular, the machine learning approach to cancer identification and tissue of origin capabilities will improve with growing datasets.

Despite a lot of promise in this data, several important shortcomings of this study need to be mentioned. First and foremost, this is a preliminary analysis of a larger collection; as such, the sample sizes are low. Additionally, the demographics are narrow and with limited demographic variety. Moreover, all the patients in the cancer cohort were already diagnosed with cancer; as such, this dataset can only demonstrate the general capability of the DEEPGEN^TM^ platform to identify cancer. On the other hand, this study addresses some shortcomings that have been discussed in previous projects [12,13,17,36]. Particularly worth mentioning is the diverse control cohort with individuals ranging from young and very healthy study participants to hospitalized patients. Nine of the positive controls were identified with possible explanations for the finding. Two individuals had a benign tumor at the time of the liquid biopsy, which can result in a signal similar to that of cancer. It is questionable if the three positives that were diagnosed with a benign lesion later were found because of an already existing pathology at time of the liquid biopsy. Previous literature has demonstrated that acute inflammations lead to an increase in somatic mutations that are otherwise associated with cancer [37,38]. All hospitalized patients with inflammation would not have been candidates for standard of care screening. Another interesting finding is the morbidly obese individuals before bariatric surgery having positive findings because this complex disease comes with chronic inflammation and an increased cancer risk. The poor health and chronic inflammatory conditions exhibited in these “controls”, identified as “cancers” by DEEPGEN^TM^, reinforces the ultra-high sensitivity of the proprietary technology stack and opens up future research possibilities in areas of chronic inflammatory conditions that are increasingly considered to be precursors to cancer and other major diseases [39,40]. However, to find specific answers to these important questions, more global research in the field of liquid biopsy is needed.

## 5. Conclusions

Despite being at an early stage of development and there being room for improvements in areas such as blood-drawing to machine learning, the DEEPGEN^TM^ platform shows a clinically viable capability to differentiate patients with and without cancer. More systematic research is needed to define the exact clinical applications as well as its large-scale performance.

## Figures and Tables

**Table 1:** Demographic parameters.

Parameter	Control *n* = 415	All Cancer *n* = 260	Bladder *n* = 25	Prostate *n* = 29	Lung *n* = 30	Liver *n* = 27	Pancreatic *n* = 40	Colorectal *n* = 66	Breast *n* = 43
Age									
Mean (standard deviation)	54.9 (15.5)	65.4 (10.9)	68.7 (11.1)	64.9 (7.3)	64.2 (9.6)	67.5 (9.7)	66.9 (9.4)	65.2 (12.3)	61.9 (12.3)
Sex, *n* (%)									
Male	117 (28)	136 (52)	18 (72)	29 (100)	10 (33)	20 (74)	21 (52)	38 (58)	0 (0)
Female	298 (72)	124 (48)	7 (28)	0 (0)	20 (67)	7 (26)	19 (48)	28 (42)	43 (100)
Comorbidities, *n* (%)									
Alzheimer’s	1 (0.2)	1 (0.4)	0 (0.0)	0 (0.0)	0 (0.0)	0 (0.0)	0 (0.0)	0 (0.0)	1 (2)
Cardiovascular disease	27 (7)	28 (11)	8 (32)	1 (3)	3 (10)	4 (15)	4 (10)	6 (9)	2 (5)
Diabetes	31 (7)	36 (14)	4 (16)	2 (7)	2 (7)	8 (30)	8 (20)	8 (12)	4 (9)
Hypertension	75 (18)	108 (42)	18 (72)	8 (28)	12 (40)	15 (56)	18 (45)	23 (35)	14 (33)
Kidney disease	5 (1)	14 (5)	4 (16)	2 (7)	1 (3)	0 (0.0)	0 (0.0)	5 (8)	2 (5)
Obesity	40 (10)	30 (12)	8 (32)	2 (7)	5 (17)	3 (11)	0 (0.0)	5 (8)	7 (16)
Respiratory disease	18 (4)	35 (13)	7 (28)	3 (10)	9 (30)	4 (15)	4 (10)	2 (3)	6 (14)
Other	141 (34)	113 (43)	18 (72)	17 (59)	16 (53)	18 (67)	12 (30)	14 (21)	18 (42)
None	185 (45)	30 (12)	1 (4)	3 (10)	3 (10)	1 (4)	3 (8)	10 (15)	9 (21)
Unknown	32 (8)	46 (18)	0 (0.0)	2 (7)	1 (3)	5 (19)	13 (32)	25 (38)	0 (0.0)
Risk Factors, *n* (%)									
Family History *	182 (44)	100 (38)	4 (16)	8 (28)	14 (47)	9 (33)	14 (35)	25 (38)	26 (60)
Smoking History	132 (32)	110 (42)	11 (44)	11 (38)	23 (77)	13 (48)	15 (38)	25 (38)	12 (28)
Medical Condition	57 (14)	65 (25)	7 (28)	11 (38)	5 (17)	8 (30)	12 (30)	18 (27)	4 (9)
Other	22 (5)	47 (18)	10 (40)	8 (28)	5 (17)	14 (52)	3 (8)	5 (8)	2 (5)
None	104 (25)	45 (17)	8 (32)	3 (10)	2 (7)	2 (7)	3 (8)	16 (24)	11 (26)
Unknown	51 (12)	23 (9)	4 (16)	4 (14)	0 (0)	5 (19)	6 (15)	3 (5)	1 (2)

* Across 4 generations (grandparents to children).

**Table 2:** Number of cancer samples and cancer finding/sensitivities at 95% and 99% specificity.

Model	Number of Cancer Samples	Cancer Findings at 95% Specificity			Cancer Findings at 99% Specificity		
	*n*	Correctly Identified Cancers, *n*	Sensitivity, %	95% Confidence Interval, Lower Bound, Upper Bound	Correctly Identified Cancers, *n*	Sensitivity, %	95% Confidence Interval, Lower Bound, Upper Bound
All cancers	260	150	57	52, 64	111	43	37, 49
Bladder	25	20	80	64, 96	8	32	14, 50
Breast	43	13	30	16, 44	7	16	5, 27
Colorectal	66	28	42	30, 54	18	27	16, 38
Liver	27	17	63	45, 81	11	41	22, 60
Lung	30	20	67	50, 84	16	53	35, 71
Pancreas	40	21	52	37, 67	15	38	23, 53
Prostate	29	21	72	56, 88	18	62	44, 80

## Data Availability

The data presented in this study are available on request from the corresponding author. The data are not publicly available due to privacy and ethical restrictions.
